# Extracellular vesicles: emerging paradigms in bovine embryo-maternal communication

**DOI:** 10.1590/1984-3143-AR2024-0065

**Published:** 2024-08-26

**Authors:** Rosane Mazzarella, Yulia Nathaly Cajas, Maria Encina Gonzalez Martínez, Dimitrios Rizos

**Affiliations:** 1 Department of Animal Reproduction, National Institute for Agricultural and Food Research and Technology, Spanish National Research Council - INIA-CSIC, Madrid, Spain; 2 Department Agrarian Production, Technical University of Madrid –UPM, Madrid, Spain; 3 Departamento de Ciencias Biológicas, Universidad Técnica Particular de Loja – UTPL, Loja, Ecuador; 4 Department of Anatomy and Embryology, Veterinary Faculty of the Complutense University of Madrid - FV-UCM, Madrid, Spain

**Keywords:** pre-implantational embryo development, reproductive fluids, extracellular vesicles, reproductive technologies

## Abstract

The oviduct and uterus provide an optimal environment for early embryo development, where effective communication between the embryo and the maternal reproductive tract is crucial for establishing and maintaining pregnancy. Oviductal and uterine-derived EVs play pivotal roles in this maternal-embryonic communication and in facilitating early embryo development. However, despite the ability of in vitro culture methods to produce viable embryos, the lack of exchange between the embryo and the mother often results in lower-quality embryos than those derived in vivo. Therefore, there is a pressing need to increase our understanding of the physiological mechanisms underlying embryo interaction with the oviduct and endometrium through EVs and to develop models capable of mimicking the in vivo environment. This review aims to provide up-to-date insights into the communication between the mother and pre-implantation bovine embryo, exploring their applications and perspectives in the field.

## Introduction

Effective communication between the embryo and the maternal reproductive tract is essential for establishing and maintaining pregnancy. Although early bovine embryo development can occur in vitro and produce viable embryos, the lack of exchange between the embryo and the mother results in lower-quality embryos compared to those in vivo-derived (Rizos et al., 2002a, b, c). In vitro-produced embryos exhibit modifications in gene expression (Lonergan et al., 2003; Rizos et al., 2002c), elevated lipid levels (Barceló‐Fimbres and Seidel, 2007; Rizos et al., 2002a), decreased tolerance to cryopreservation (Rizos et al., 2008), altered metabolic pathways (Khurana and Niemann, 2000), and decreased success rates in establishing pregnancy (Pontes et al., 2009). These findings highlight the crucial role of the oviduct and uterus in supporting embryo development and influencing embryo quality, with implications for successful pregnancy outcomes.

The oviduct and uterus provide an optimal microenvironment for pre-implantation bovine embryo development. Within the oviduct, the early embryo undergoes its first divisions or cleavages, metabolic and energetic changes, and through minor and major embryo genomic activation (EGA) around the four-cell and eight to 16-cell stages, respectively (Graf et al., 2014; Lonergan and Forde, 2014). The bovine embryo develops in the oviduct for approximately three and a half to four days before migrating to the uterus via the utero-tubal junction. The pre-implantation embryo continues developing in the uterus through the morula, where individual blastomeres can no longer be distinguished, leading to blastocyst formation on days six to eight. During this process, there is a growth of the blastocoel and the first cellular differentiation process, forming the inner cell mass, which gives rise to the fetus, and the trophoblast, which develops into the fetal placenta (Frankenberg et al., 2016). In this context, embryo development is supported by substances originating from the oviductal epithelium and the uterine endometrium within their fluids, thus providing the embryo with the necessary physiological and biochemical conditions (Li and Winuthayanon, 2017). Among the components of the oviductal (OF) and uterine fluids (UF) are the extracellular vesicles (EVs).

EVs, lipid bilayer-delimited nanoparticles, are actively secreted by cells into the extracellular environment (Raposo and Stoorvogel, 2013). EVs are categorized into exosomes, microvesicles, and apoptotic bodies based on their size, biogenesis, and secretion (György et al., 2011). Exosomes, the most studied population of EVs, are small EVs with diameters ranging from 30 to 150 nm (Raposo and Stoorvogel, 2013; Théry et al., 2018). Their biosynthesis begins with multivesicular bodies (MVBs) forming via plasma membrane endocytosis. Subsequently, intraluminal vesicles (ILVs) are generated within MVBs, which are released into the extracellular space (Théry et al., 2002). These vesicles play crucial roles in regulating recipient cells and facilitating cell-to-cell communication through the transfer of their bioactive materials such as proteins (Simpson et al., 2008), lipids (Subra et al., 2007), and various nucleic acids, including messenger RNAs (mRNAs) and non-coding RNAs, notably microRNAs (miRNAs) (Jeppesen et al., 2019; Valadi et al., 2007). The diverse cargo carried by EVs reflects their potential to mediate physiological and pathological processes, making them significant entities in paracrine and autocrine cellular signaling networks (Yuana et al., 2013). In recent years, EVs have gained recognition for their remarkable potential as biomarkers and integral components of maternal-embryonic communication.

EVs have been recognized as significant constituents of OF, UF, and are also secreted by embryos. Oviductal fluid-derived EVs (OF-EVs), or oviductosomes, and uterine fluid-derived EVs (UF-EVs), or uterosomes, are believed to play roles in maternal communication with gametes and embryos (Cajas et al., 2021). Functionally, OF-EV (Almiñana et al., 2017; Lopera-Vasquez et al., 2017), UF-EVs (Leal et al., 2022), and also EVs from the bovine oviductal epithelial cells (BOECs) (Lopera-Vásquez et al., 2016) conditioned culture medium are internalized by bovine embryos and improve *in vitro* embryo development and quality. Additionally, the sequential use of EVs from OF and UF during bovine embryo in vitro culture (IVC) also improved embryo quality by increasing cell numbers and lowering lipid contents in blastocysts (Leal et al., 2022). These studies, along with others discussed here, underscore the potential of EVs in enhancing embryo quality by facilitating communication between the embryo and the maternal environment. Additionally, it emphasizes the need for a deeper understanding of these physiological mechanisms and for developing models that facilitate the exploration of maternal-embryonic crosstalk in both in vivo and in vitro settings. Therefore, this review offers current insights into the communication between the mother and the pre-implantation bovine embryo, examining their applications and prospects in the area.

## In vivo derived EVs from the female reproductive tract

### Embryo-maternal communication through OF-EVs

**Figure 1:**
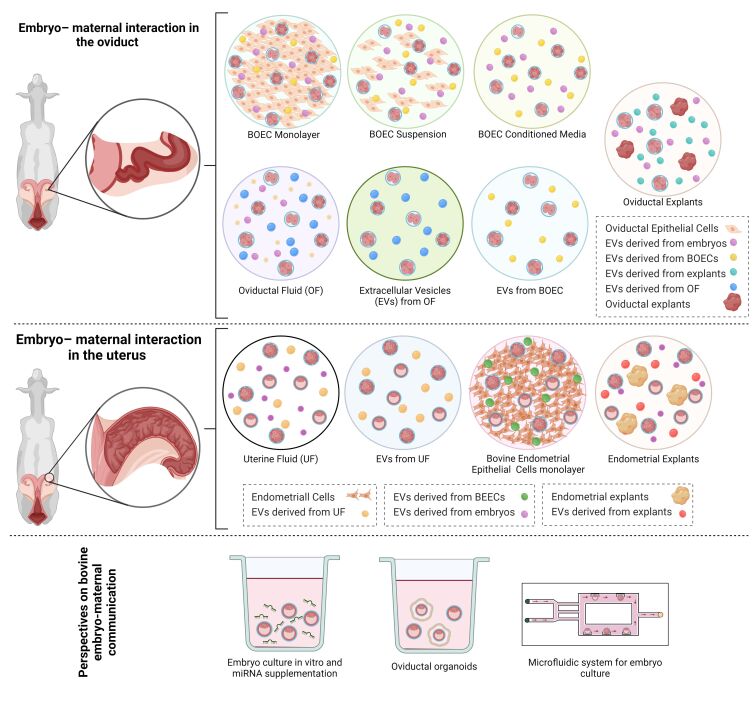
Schematic representation of embryo–maternal interaction mediated by oviductal and uterine EVs in vitro and in vivo, and perspectives on studying this communication in vitro. Figure created with BioRender.com.

After fertilization, the embryo’s growth and division are sustained by substances released by the oviductal epithelium and present in the OF. Oviductal EVs are recognized as key components of OF, with potential roles in mediating gamete and maternal interactions (Bastos et al., 2022). In cattle, OF-EVs and their role in maternal embryonic communication have been studied by different groups, as shown in Table 1 and Figure 1.

**Table 1:** Summary of main findings in bovine in vivo and in vitro early embryo-maternal communication by EVs.

**Topic**	**EV source**	**Findings**	**EV isolation method**	**EV markers**	**EV content**	**Reference**
Embryo-maternal interactions through EVs **IN VIVO**	Oviductal fluid	Isthmus-derived EVs enhance embryo quality and induce significant differences in gene expression related to metabolism, epigenetics, and water channel traffic.	UC	CD9, TSG101 and ERM (WB)	-	(Lopera-Vasquez et al., 2017)
		EVs were internalized by the embryo during in vitro culture and enhanced their ability to reach the blastocyst stage.	UC	HSP70 (WB)	Proteins	(Almiñana et al., 2017)
		Changes in EV cargo across the estrous cycle are potentially related to gamete/embryo-oviductal interactions.	UC	HSP70 (WB)	mRNAs, ncRNAs, and proteins	(Almiñana et al., 2018)
		The metabolite content of EVs is regulated across the estrous cycle in cattle.	UC	CD81 and HSP70 (WB)	Metabolites	(Gatien et al., 2019)
		OF-EVs cargo regulates the embryonic transcriptome.	-	-	mRNAs and miRNAs	(Bauersachs et al., 2020)
		The embryo presence modulates miRNA contents of EVs and BOECs in vivo.	UC	-	miRNAs	(Mazzarella et al., 2021)
		Protein signature in pregnant animals' OF-EVs suggests interactions between the mother and the embryo through EVs in the oviduct.	SEC	CD63, CD81, and CD44 (FC)	Proteins	(Mazzarella et al., 2023b)
	Oviductal fluid and Uterine Fluid	miRNA profile changes in OF- and UF-EVs across the estrous cycle suggest miRNAs' role in cell signaling and reproductive functions.	UC	CD9 and HSP70 (WB)	miRNAs	(Hamdi et al., 2021)
		OF- and UF-EVs in sequential IVC enhanced blastocyst quality, survival rate, and lipid content and changed lipid metabolism gene expression.	UC	CD9 and HSP70 (WB)	miRNAs	(Leal et al., 2022)
		OF- and UF-EVs miRNA cargo may influence embryo development and quality and potentially regulate immune response and implantation.	-	-	miRNAs	(Mazzarella et al., 2024)
	Uterine Fluid	UF-EVs contain IFNT on days 17, 20, and 22 of pregnancy and can up-regulate apoptosis-related genes and adhesion molecules in BEECs.	Exo-Quick®	CD63 and HSP70 (WB)	Proteins	(Kusama et al., 2018)
		In vitro culture supplementation with UF-EVs in nuclear transfer embryos significantly increased blastocyst formation rates.	UC	CD9 (WB)	-	(Qiao et al., 2018)
		The presence of multiple D7 embryos in the uterus can lead to significant changes in the protein and miRNA contents of UF-EVs.	Exo-Quick^TC^	HSP70, CD63, RAB5, among others (WB)	Proteins and miRNAs	(Kusama et al., 2021)
		Changes in UF-EV concentration and protein profile at follicular and luteal phases suggest EV modulation of uterine microenvironment across the estrous cycle.	SEC	ANXA4, CD63, CD1, among others (MS)	Proteins	(Piibor et al., 2023)
		The presence of an embryo modulates the uterine environment, specifically the protein profile within UF-EVs.	SEC	CD63, CD81, and CD44 (FC)	Proteins	(Mazzarella et al., 2023a)
		OF-EVs increase embryo diameter and IFNT expression.	ExoLutE®	CD9, CD63, CD81 and CD40 (FC)	-	(Aguilera et al., 2024)
Embryo-maternal interactions through EVs **IN VITRO**	BOECs	BOECs-CM and BOECs-EVs improve embryo quality (number of cells and gene expression) and cryosurvival.	UC	CD9, CD63, TSG101 and ERM (FC)	Proteins	(Lopera-Vásquez et al., 2016)
		In vivo (OF) and in vitro-derived (BOECs) EVs have different secretion/content.	UC	HSP70 (WB)	-	(Almiñana et al., 2017)
	Oviductal explants	Oviductal explants-EVs and OF-EVs share common proteins involved in early embryo development.	SEC	CD63, CD81, and CD44 (FC)	Proteins	(Mazzarella et al., 2023c)
	BEECs	BEECs-EVs improve the pre-implantation development of in vitro-produced embryos.	ExoLutE®	CD9, CD63, CD81 and CD40 (FC)	-	(Aguilera et al. 2024)
**EMBRYO DERIVED EVS**		Blastocysts secrete EVs into the culture medium, and these EVs vary depending on embryo competence.	UC	CD63 and CD9 (FC)	-	(Mellisho et al., 2017)
		Bovine somatic cell nuclear transfer (SCNT) embryos secrete EVs, which are essential for the subsequent development of these embryos.	UC	CD9 (IHC)	-	(Qu et al., 2017)
		Embryonic EVs act as autocrine embryotropins in embryo–embryo communication during in vitro embryo culture.	OptiPrep™	CD63 and CD9 (WB)	-	(Pavani et al., 2019)
		Individually cultured embryos secrete EVs that may indicate their developmental competency.	SEC	CD9, CD63, CD81, among others (MA)	-	(Dissanayake et al., 2020)
		EVs released by individually cultured bovine embryos impact gene expression in BOECs based on the quality of the embryo.	SEC	-	-	(Dissanayake et al., 2021)
		In vivo and in vitro‐produced bovine hatched blastocysts (Day 9) secret EVs with different miRNA profiles.	Exo-Quick^TC^	-	miRNAs	(Bridi et al., 2021)
		EVs from in vitro- and in vivo-produced embryos have different effects on the transcriptomic profiles of IFNT-stimulated genes in BEECs.	ExoLutE®	CD9, CD63, CD81 and CD40 (FC)	-	(Aguilera et al., 2023)
		EVs from in vitro-produced embryos induce non-classical ISGs in BEECs	ExoLutE®	CD9, CD63, CD81 and CD40 (FC)	-	(Aguilera et al., 2024)

EVs: Extracellular vesicles; UF: Uterine fluid; OF: Oviductal fluid; BOEC: bovine oviductal epithelial cells; BEEC: bovine endometrial epithelial cells; CM: conditioned medium; UC: Ultracentrifugation; SEC: Size exclusion chromatography; WB: Western blotting; FC: flow cytometry; IHC: Immunohistochemistry; MA: Microarray; IFNT: Interferon tau.

Oviductal EVs from different oviductal regions (i.e., ampulla and isthmus) and sources (i.e., in vivo vs. in vitro) have distinct compositions and contrasting effects on embryo development. EVs from the isthmus have a beneficial effect on embryo quality, reflected by enhanced cryotolerance, compared to EVs from the ampulla, thereby establishing a correlation between the oviductal environment and embryo development (Lopera-Vasquez et al., 2017). Although specific contents of these EVs were not analyzed, the findings suggest potential differences in isthmus-derived contents that may benefit embryos more significantly. Furthermore, Almiñana et al. (2017) compared the protein cargo of EVs derived from in vivo sources, specifically OF-EVs, and those derived from in vitro sources obtained from the medium conditioned by BOECs. Differentially abundant proteins were involved in sperm-oocyte binding, fertilization, and embryo development, reinforcing OF-EVs’ role in gamete/embryo-oviduct interactions. Additionally, specific proteins, such as oviductal specific glycoprotein 1 (OVGP1), were lacking in in vitro EVs.

The oviductal environment and its epithelial cells are under hormonal regulation (Binelli et al., 2018), which also influences the composition of the EV content. Almiñana et al. (2018) investigate the OF-EVs content of protein, mRNA, and small RNA levels across the estrous cycle. Their results indicate a dynamic molecular profile under hormonal control, with clear differences between post- and pre-ovulatory stages. Furthermore, Hamdi et al. (2021) described changes in miRNA profile and abundance across different estrus cycle stages. Notably, eight miRNAs exhibited increased expression from stage 1 (day 1 to 4 after ovulation) to stage 4 (day 17 to 21) and are associated with cell signaling, intercellular junctions, and reproductive functions. Moreover, the findings suggest that miRNAs within EVs may play a dual role, contributing to maternal-embryonic and autocrine communication within oviductal cells, thus preparing the oviduct for gamete/early embryo reception.

It has also been shown that pregnancy can induce alterations in the content of OF-EVs. Mazzarella et al. (2021) findings suggest that pregnancy induces modulation in the miRNA contents of OF-EVs, along with alterations in the levels of miRNAs and mRNAs in BOECs. The functional analysis of miRNAs differently expressed in OF-EVs emphasizes the enrichment of pathways essential for physiological functions like inflammation, cell proliferation, and immune response, which play critical roles in reproductive tract function and embryo development (Mazzarella et al., 2021). Complementary, Mazzarella et al. (2023d) described embryo-induced alteration in the protein cargo of OF-EVs from pregnant heifers on day 3.5, with differentially abundant proteins primarily associated with EGA, DNA repair, embryonic cell differentiation, migration, and immune tolerance. Although this model does not exclude a potential effect of sperm, these findings suggest that embryos may trigger changes in EV content in both heifers and cows. Moreover, these results also indicate that communication between the embryo and the maternal environment begins within the oviduct during the early stages of embryo development.

OF-EVs can be used as a tool to improve early embryo development. As previously mentioned, Lopera-Vasquez et al. (2017) and Almiñana et al. (2017) have utilized OF-EVs as a promising and effective model for enhancing embryo development and quality within in vitro environments. Moreover, EV content can differentially modulate embryonic transcriptome in embryos supplemented with OF-EVs compared to the control (Bauersachs et al., 2020). Interestingly, the authors also observed a correlation between the mRNA and miRNA content of OF-EVs and the gene alterations observed in those embryos. Recently, our group reported that sequentially supplementation of in vitro culture medium with OF-EVs (day 1 to 4) followed by UF-EVs (day 4 to 8) could improve embryo development and quality by modulating genes related to lipid metabolism, reducing lipid content, increasing blastocyst cell number, and improving cryotolerance (Leal et al., 2022). Bioinformatic analyses suggest that these results are potentially mediated by the miRNA content found in OF- and UF-EVs (Mazzarella et al., 2024). Therefore, miRNAs within both OF- and UF-EVs are potentially involved in maternal-embryonic communication within the oviduct and uterus.

### Embryo-maternal communication through UF-EVs

The endometrium produces and releases a variety of compounds into the uterine cavity known as histotroph (Bazer et al., 2011). These compounds consist of embryotrophic factors, which, in response to progesterone, IFNT, and other stimuli, promote blastocyst growth and survival, conceptus elongation, and implantation (Spencer et al., 2017). EVs have been identified as a component of UF, providing a novel means of communication between the developing conceptus and the uterine endometrium (O’Neil et al., 2020). Few research groups have investigated UF-EVs derived from bovine sources, as shown in Table 1 and Figure 1.

The composition of UF-EV cargo undergoes dynamic changes across the estrous cycle. Hamdi et al. (2021) reported that the miRNA profile of UF-EVs dynamically changes throughout the estrous cycle. Significant differences were observed between stage 1 (day 1 to 4 after ovulation) and stage 3 (day 11 to 17), with 11 miRNAs showing higher abundance in stage 3. This suggests a potentially increased level of transcriptional activity during this stage, which aligns with maternal recognition of pregnancy and conceptus elongation. Furthermore, it has been reported that there is variation in the proteomic profiles of UF-EVs among the follicular and luteal phases of the estrous cycle (Piibor et al., 2023). The differences observed in the protein cargo suggest the influence of EVs on the uterine environment, particularly in assisting with endometrial cell polarity and remodeling during the estrous cycle.

The composition of UF-EV cargo is also affected by the embryo’s presence. Kazuya Kusama et al. (2021) reported alterations in the miRNA profile of UF-EVs on day 7 of pregnancy, suggesting their potential role in mediating innate immunological interactions. (Kusama et al., 2021). Recently, our group analyzed EVs’ protein cargo from the UF of pregnant and cyclic heifers’ on Day 7 to identify potential mediators of maternal-embryonic communication in cattle. The presence of a single embryo could induce changes in the EVs protein cargo, with proteins exclusive to pregnant heifers being associated with pathways such as signal transduction, cellular processes, the endocrine system, metabolism, and the immune system (Mazzarella et al., 2023c). Furthermore, proteins associated with embryo development, identified in pregnant UF-EVs, potentially contribute to maternal-embryonic crosstalk by modulating blastocyst cell cycle progression, cell polarity, and inner cell mass proliferation within the uterine environment (unpublished data).

When working with pregnant animals, it is essential to consider that the EVs found in UF can originate from both endometrial cells and the embryo. For example, Kazuya Kusama et al. (2018) investigated the protein content of EVs during the peri-implantation period on days 17, 20, and 22 of pregnancy. Among the identified proteins was the pregnancy recognition factor IFNT, indicating that they originated from the conceptuses. Moreover, the same study also showed that supplementation of EVs on bovine endometrial epithelial cells (BEECs) upregulated the cell expression of apoptosis-related genes and adhesion molecules, suggesting EVs participation in conceptus implantation.

UF-EVs can be used as a tool to improve early embryo development. The supplementation of the IVC medium with UF-EVs significantly increases blastocyst and hatching rates, enhancing the developmental competence of somatic cell nuclear transfer embryos (Qiao et al., 2018). Moreover, as previously mentioned, sequential supplementation of the IVC with OF-EVs followed by UF-EVs demonstrated the influence of EVs on modulating lipid metabolism genes and improving embryonic development and quality (Leal et al., 2022). Furthermore, functional enrichment analysis of the miRNA content within UF-EVs underscored their potential involvement in regulating genes related to embryo lipid metabolism and endometrial receptivity (Mazzarella et al. 2024). These studies demonstrate that UF-EVs are potentially involved in maternal-embryonic communication and successful implantation

## In vitro-derived EVs from the bovine female reproductive tract

Different in vitro models have been employed to explore the oviductal and uterine environments and their roles in embryo-maternal communication. Within the oviduct, BOECs have been studied in various forms, including monolayer, suspension, air-liquid interphase, three-dimensional (3D) cultures, and microfluidics approach (Ferraz et al., 2018), as well as using BOEC-conditioned media (Lopera-Vásquez et al., 2016) and bovine oviductal explants (Mazzarella et al., 2023e). Similarly, in the uterus, bovine endometrial epithelial cells (BEECs) monolayer (Sponchiado et al., 2020), microfluidics approach (De Bem et al., 2021), and endometrial explants (Passaro et al., 2018) have been used for the same purpose. However, there is limited research on EVs in the spent medium, their cargo, and their effects on early embryo development.

BOECs and BEECs cultures offer an easy and well-established method for assessing cellular function and obtaining and studying EVs released in their spent media in vitro. BOECs have mainly been utilized to aid in vitro embryo development. Notably, EVs from BOECs conditioned culture medium are internalized by bovine embryos and favored embryo development and the quality of the produced blastocysts in vitro in terms of cryotolerance (Lopera-Vásquez et al., 2016). However, it is essential to acknowledge that EVs derived from in vivo (OF-EVs) and in vitro (BOECs) sources display distinct protein cargo, which is a limitation to the use of in vitro-derived EVs in investigating maternal-embryonic communication (Almiñana et al., 2017). Indeed, recent studies have indicated that EVs from in vivo (UF-EVs) and in vitro (BEECs) sources exert different effects on in vitro embryo development, with UF-EVs demonstrating superior effects on embryo quality (Aguilera et al., 2024).

The use of explants allows the culture of all cell types and structures present in vivo. Preliminary data from our group suggests that both oviductal and uterine explants respond to embryos as early as the 8-16 cell stage and to blastocysts, respectively. Notably, EVs from oviductal and uterine explants exhibit distinct protein profiles when explants are cultured alone or in the presence of embryos. Although differences in protein cargo of oviductal EVs in vivo (OF-EVs) and in vitro (oviductal explants) have been identified, EVs from oviductal explants co-cultured with embryos and OF-EVs from pregnant heifers share common proteins involved in early embryo development (Mazzarella et al., 2023e). This finding supports embryo-maternal communication via EVs and highlights the utility of ex vivo models for studying this process.

## EVs derived from bovine embryos

Embryonic-derived EVs facilitate bidirectional communication between the pre-implantation embryo and the mother (Cajas et al., 2021). Notably, EVs from bovine embryos have been detected as early as day 2 of the development (Dissanayake et al., 2020). Additionally, these EVs are involved in autocrine signaling, fostering communication among embryos cultured in vitro (Qu et al., 2017). For instance, Qu et al. 2017 demonstrated that supplementing culture media with embryonic EVs enhances blastocyst formation and quality in SCNT embryos, suggesting that embryos’ EVs impact their growth and function within the same in vitro environment.

The secretion and content of embryonic EVs vary depending on the origin of the bovine embryo. For example, embryos derived from fertilization or parthenogenetic processes release distinct quantities of EVs (Mellisho et al., 2017). Moreover, embryos from in vivo and in vitro origins yield EVs with distinct miRNA profiles (Bridi et al., 2021). Furthermore, although EVs from both origins (in vivo and in vitro) activate classical and non-classical IFNT signaling pathways, they induce different gene expression patterns in BEECs (Aguilera et al., 2023). These results indicate that distinct communication occurs between the embryo and mother based on the embryo’s origin, potentially affecting pregnancy establishment and maintenance.

Furthermore, studies have shown that EV concentrations and contents in culture media vary depending on the embryo’s developmental stage and competence (Mellisho et al., 2017). For example, on day 8 of IVC, embryonic EVs isolated from the conditioned medium by degenerating embryos exhibit higher concentrations and smaller diameters than those from high-quality blastocysts (Dissanayake et al., 2020). Additionally, high- and low-quality embryos release embryonic DNA fragments within EVs in their culture medium, suggesting a potential indicator of embryo quality (Caamaño et al., 2024). However, further investigations are required to determine if specific DNA fragments are characteristic of either high or low-quality embryos. Consequently, EVs in the culture medium of in vitro embryos hold promise as non-invasive indicators of embryo quality.

## Perspectives on bovine embryo-maternal communication

Since the discovery of EVs as mediators of embryo-maternal communication, extensive research has investigated this interaction both in vivo and in vitro. However, challenges remain in understanding and replicating this communication in vitro. Innovative in vitro models, such as microfluidic approaches and 3D organoid culture systems, have been increasingly utilized to mimic the maternal in vivo environment more accurately (Thompson et al., 2022). For example, Ferraz et al. (2018) integrated 3D printing and microfluidics in an “oviduct-on-a-chip” platform. Although the resulting bovine zygotes resembled those produced in vivo, improvements are needed for this technology as the success rates were lower than those of traditional in vitro methods (Ferraz et al., 2018). More recently, bovine oviductal organoids have been characterized, showcasing the efficacy of the technology (Lawson et al., 2023). Nevertheless, the impact of gametes and embryos on these organoids has yet to be studied. Although these methods appear promising, there is still a lack of knowledge and characterization of the EVs produced and exchanged in this environment.

Moreover, EV cargo identified in reproductive fluids, such as miRNAs, can serve as new biomarkers and tools for improving assisted reproductive technologies. For instance, bta-mir-133b and bta-mir-483, exclusively present in OF-EVs from pregnant cows (Mazzarella et al., 2021), are taken up by embryos when added to IVC (Cañón-Beltrán et al., 2023; Mazzarella et al., 2023a). Although they do not affect cleavage and blastocyst rates, miR483-3p enhances blastocysts’ mitochondrial activity and decreases lipid content, suggesting its role in pre-implantation embryo-maternal interaction (Mazzarella et al., 2023b). Furthermore, bta-mirR-148b, upregulated on OF-EVs, when added in the IVC system, enhances embryo quality and modulates the TGF-β signaling pathway (Cañón-Beltrán et al., 2024). These findings emphasize the importance of understanding the functional effects of EV cargo from maternal fluid on embryo development and quality.

## Conclusion

Oviductal and uterine-derived EVs play critical roles in maternal-embryonic communication and early embryo development, undergoing dynamic changes in cargo influenced by embryo presence. Both OF- and UF-EVs enhance early embryo development and quality in vitro, underscoring the importance of studying their cargo. While in vitro models have been utilized to mimic this communication, it is essential to acknowledge that EVs from BOEC and BEEC cultures and oviductal and uterine explants exhibit distinct contents compared to in vivo-derived EVs. This emphasizes the need for innovative in vitro models like 3D organoids and microfluidic approaches to better simulate the in vivo microenvironment. Additionally, comprehensive studies of EV cargo generated by these models are necessary. Further research into EVs derived from in vitro-produced embryos and the development of quality biomarkers is also crucial. In conclusion, advancing in vitro models to mimic the in vivo microenvironment will enhance our understanding of physiological maternal-embryonic communication via EVs and improve the efficiency of current in vitro embryo production systems.
